# Numerical Method Using Cubic B-Spline for a Strongly Coupled Reaction-Diffusion System

**DOI:** 10.1371/journal.pone.0083265

**Published:** 2014-01-10

**Authors:** Muhammad Abbas, Ahmad Abd. Majid, Ahmad Izani Md. Ismail, Abdur Rashid

**Affiliations:** 1 Department of Mathematics, University of Sargodha, Sargodha, Pakistan; 2 School of Mathematical Sciences, Universiti Sains Malaysia, Penang, Malaysia; 3 Department of Mathematics, Gomal University, Dera Ismail Khan, Pakistan; University of Calgary, Canada

## Abstract

In this paper, a numerical method for the solution of a strongly coupled reaction-diffusion system, with suitable initial and Neumann boundary conditions, by using cubic B-spline collocation scheme on a uniform grid is presented. The scheme is based on the usual finite difference scheme to discretize the time derivative while cubic B-spline is used as an interpolation function in the space dimension. The scheme is shown to be unconditionally stable using the von Neumann method. The accuracy of the proposed scheme is demonstrated by applying it on a test problem. The performance of this scheme is shown by computing 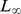 and 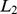 error norms for different time levels. The numerical results are found to be in good agreement with known exact solutions.

## Introduction

This study is concerned with the numerical solution of strongly coupled reaction-diffusion system using cubic B-spline. Reaction-diffusion system arises in the study of biology, chemistry and population dynamics. It can be used to describe a mathematical model of a class of chemical exchange reaction that arises in the transport of ground water in an aquifer [1]. The mathematical formulation of the problem is:
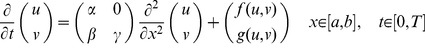
(1)with the initial conditions
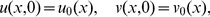
(2)and the boundary conditions
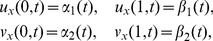
(3)where 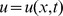 and 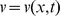 are the concentrations of the two substances in the interaction and the constants 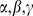 are such that 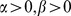, 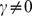. The following consistency conditions hold
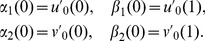
(4)The global solutions of such a system of equations have attracted the attention of several researchers [2]–[6]. Researchers have also investigated the existence, uniqueness and boundedness of the global solution in bounded and unbounded region [3], [6]. Cao and Sun [1] derived a finite difference scheme by the method of reduction of order for the numerical solution of strongly coupled reaction-diffusion system with Neumann boundary values conditions. They proved the solvability and convergence by using the energy method. Several researchers focused on analytical solutions of nonlinear equations by using approximate analytical methods. Examples include Ghoreishi et al [7] who obtained the analytical solution for a strongly coupled reaction-diffusion system by using the Homotopy Analysis Method. The solution of the system was calculated in the form of an infinite series with easily computed components. This method cannot always guarantee the convergence of approximate series [8], [9] and the method depends on choosing the proper linear operator and initial guesses.

The study of spline functions is a key element in computer aided geometric design [10] and also several other applications. It has also attracted attention in the literature for the numerical solution of linear and non-linear system of second-order boundary value problems [11], [12] that arise in science and engineering. Some researchers have considered spline collocation method for diffusion problems [15]–[19]. Advection-diffusion equation arises frequently in the study of mass, heat, energy and vorticity transfer in engineering. Bickley [13] introduced the idea of using a chain of low-order approximation (cubic splines) rather than a global high-order approximation to obtain better accuracy for a linear ordinary differential equation. Fyfe [14] used the method proposed by Bickley [13] and conducted an error analysis. It was concluded that the spline method is better than the usual finite difference scheme because it has the flexibility to obtain the solution at any point in the domain with greater accuracy. The numerical solution of some partial differential equations can be obtained using B-spline functions of various degrees which can provide simple algorithms. As an example, a combination of finite difference approach and cubic B-spline method was used to solve the Burgers' equation [15], heat and wave equation [16], [17], advection-diffusion equation [18] and coupled viscous Burgers' equation [19].

Sahin [24] presented the B-spline methods in several degrees for the solution of following non linear reaction-diffusion system
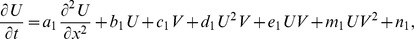


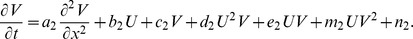
The finite element method was employed for the solution of reaction-diffusion systems and the time discretization of the system was achieved by the using Crank-Nicolson formulae. The system was considered only reaction-diffusion but the problem we propose to investigate is a strongly reaction-diffusion system with an extra term 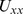 in the second equation of system (1).

In this paper we aim to apply the combination of finite difference approach and cubic B-spline method to solve the system (1). Some researchers have utilized the B-spline collocation methods to solve systems of differential equations but so far as we are aware not the system (1). A usual finite difference scheme is used to discretize the time derivative. Cubic B-spline is applied as an interpolation function in the space dimension. The unconditional stability property of the method is proved by von Neumann method. The feasibility of the method is shown by a test problem and the approximated solutions are found to be in good agreement with the exact solution.

This paper is structured as follows: Firstly, we discuss a numerical method incorporating a finite difference approach with cubic B-spline. Secondly, the von Neumann approach is used to prove the stability of method and compare the numerical solution with exact solution of system (1). Thirdly, a test problem is considered to show the feasibility of the proposed method. Finally, the conclusion of this study is given.

## Description of Cubic B-Spline Collocation Method

In this section, we discuss the cubic B-spline collocation method for solving numerically the strongly coupled reaction-diffusion system (1). The solution domain 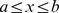 is equally divided by knots 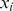 into 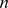 subintervals 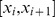, 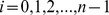 where 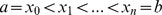. Our approach for strongly coupled reaction-diffusion system using collocation method with cubic B-spline is to seek an approximate solution as [20]

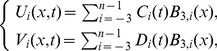
(5)where 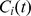 and 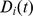 are (time dependent) quantities which are to be determined for the approximated solutions 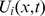 and 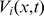 to the exact solutions 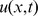 and 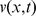 respectively, at the point 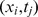 and 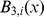 are cubic B-spline basis functions which are defined by [21]

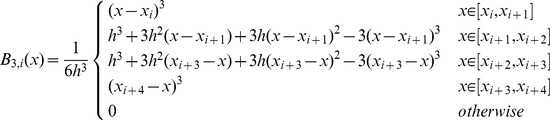
(6)where 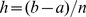. The approximations 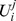 and 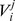 at the point 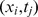 over subinterval 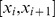 can be defined as
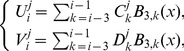
(7)where 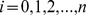. So as to obtain the approximations to the solutions, the values of 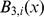 and its derivatives at nodal points are required and these derivatives are tabulated in Table 1.

**Table 1:** Values of 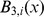 and its derivatives.

	*x_i_*	*x_i_*_+1_	*x_i_*_+2_	*x_i_*_+3_	*x_i_*_+4_
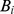	0	1/6	2/3	1/6	0
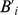	0	1/2*h*	0	−1/2*h*	0
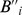	0	1/*h* ^2^	−2/*h* ^2^	1/*h* ^2^	0

Using approximate functions (6) and (7), the values at the knots of 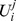 and 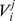 and their derivatives up to second order are determined in the terms of time parameters 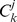 and 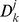 as
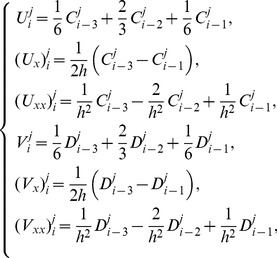
(8)The approximations of the solutions of the system (1) at 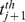 time level can be given by as [22]

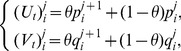
(9)where 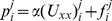, 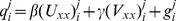 and 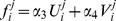, 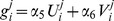, where 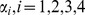 are constants and the subscripts 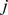 and 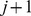 are successive time levels, 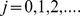. Discretizing the time derivatives in the usual finite difference way and rearranging the equations, we obtain
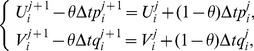
(10)where 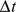 is the time step. It is noted that the system becomes an explicit scheme when 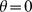, a fully implicit scheme when 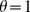, and a Crank-Nicolson scheme when 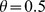 [15] with time stepping process being half explicit and half implicit. In this paper, we use the Crank-Nicolson approach. Hence, (10) becomes
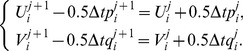
(11)The system thus obtained on simplifying (11) after using (8) consists of 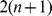 linear equations in 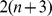 unknowns 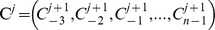, 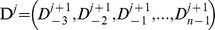 at the time level 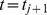. So as to obtain a unique solution to the resulting system, four additional linear equations are required. Thus, equation (7) is applied to the boundary conditions (3) to obtain
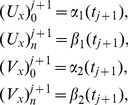
(12)From equations (11) and (12), the system becomes a matrix system of dimension 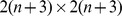 which is a bi-tridiagonal system that can be solved by the Thomas Algorithm [23]. From equation (10) and (12), the system can be written in the matrix vector form as:
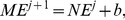
(13)where
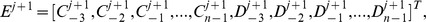


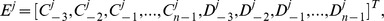


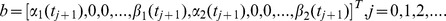
and 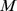 is an 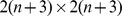 dimensional matrix given by
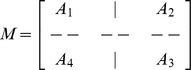


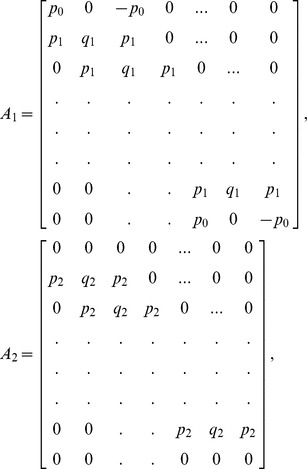


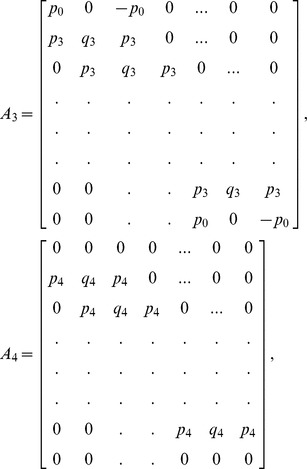


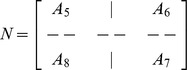


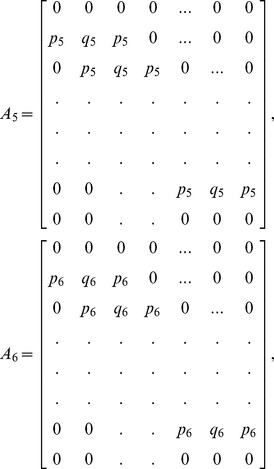


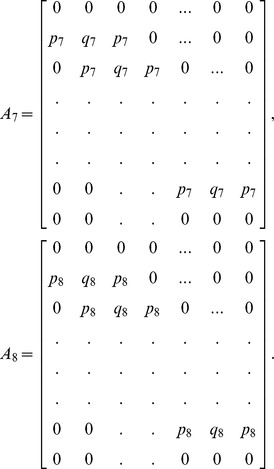



Also the entries in sub-matrices 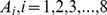 have the following form
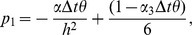


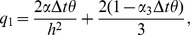


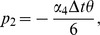


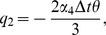


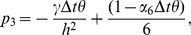


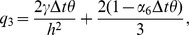


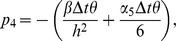


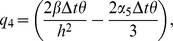


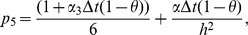


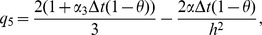


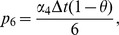


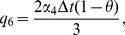


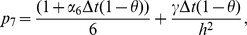


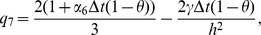


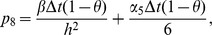


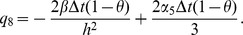



### Initial state

After the initial vectors 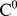 and 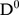 have been computed from the initial conditions, the approximate solutions 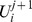 and 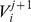 at a particular time level can be calculated repeatedly by solving the recurrence relation [19].


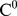
 and 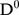 can be obtained from the initial condition and boundary values of the derivatives of the initial condition as follows [15]:
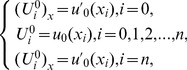
(14)and similarly for approximate solution 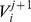


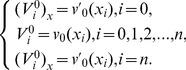
(15)Thus the equations (14) and (15) yield a 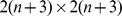 matrix system, of the form
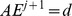
where
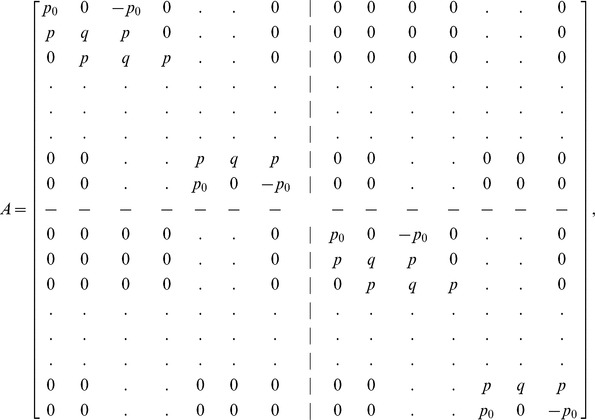


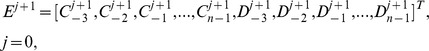


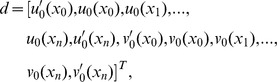
and 
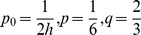
.

The solution of above system can be found by Thomas Algorithm.

### Stability of the Scheme

In this section, von Neumann stability method is applied for investigating the stability of the proposed scheme. This approach has been used by many researchers [15]–[19]. Substituting the approximate solution 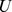 and 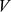, their derivatives at the knots and 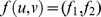, 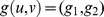 into equation (10) yields a difference equation with variables 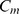 and 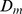 given by:
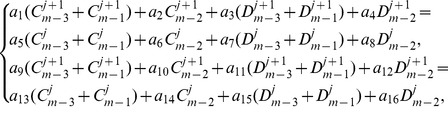
(16)where
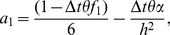


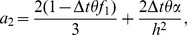


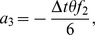


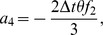


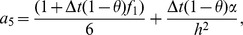


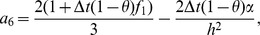


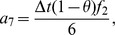


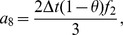


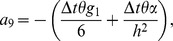


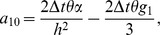


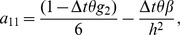


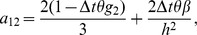


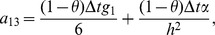


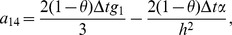


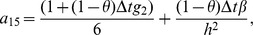


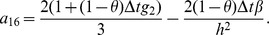



Now on inserting the trial solutions (one Fourier mode out of the full solution) at a given point 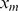 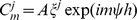 and 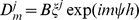 into equation (16) and rearranging the equations, where 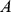 and 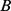 are the harmonics amplitudes, 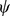 is the mode number, 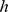 is the element size and 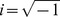 we get
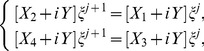
(17)where
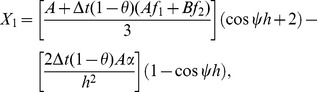


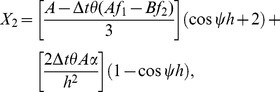


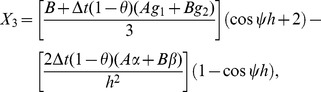


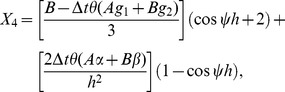


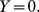



On direct calculation of equation (17) we obtain
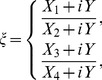
(18)For stability, the maximum modulus of the eigen-values of the matrix has to be less than or equal to one. As 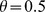 is used in the proposed scheme, we thus substitute the value of 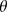 into equation (18) and after some algebraic calculation, it can be noticed that
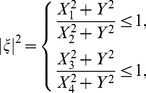
(19)Thus, from (19), the proposed scheme for strongly coupled reaction-diffusion equations is unconditionally stable since the modulus of the eigen-values must be less than one. This means that there are no constraints on grid size 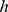 and step size in time level 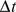 but we should prefer those values of 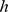 and 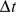 for which we obtain the best accuracy of the scheme.

## Results and Discussion

To test the accuracy of present method, one example is given in this section with 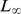 and relative 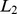 error norms are calculated by
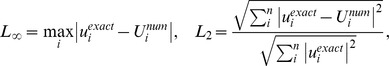
and in similar way for the numerical solution 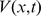.

The numerical order of convergence 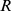 for both 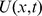 and 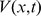 of present method is obtained by using the formula [19].
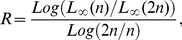
(20)where 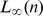 and 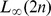 are the errors at number of partitions 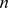 and 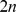 respectively.

We compare the numerical solution obtained by cubic B-spline collocation method for strongly coupled reaction-diffusion system (1) with known exact solution.

### Example 1

In this example, we present a strongly coupled reaction-diffusion system (1) with numerical solution to show the capability and efficiency of cubic B-spline collocation scheme.

We consider the following problem
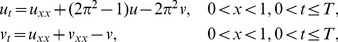
(21)with initial conditions
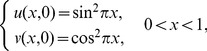
(22)and boundary conditions as follows:
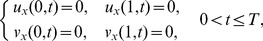
(23)It is straightforward to verify that the following are the exact solutions [1]

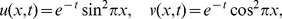
We use the cubic B-spline collocation method (11)–(12) and (14)–(15) to compute the numerical solution of (21)–(23). Table 2 and Table 3 show the acceptable comparison between numerical and exact solutions at some grid points at 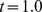 with different number of partitions.

**Table 2:** Some numerical solution of 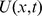 at 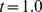.

*n*/*x*	0.125	0.25	0.375	0.625	0.75	0.875
16	0.09166515	0.18393918	0.27621321	0.27621321	0.18393918	0.09166515
32	0.06452148	0.18393899	0.30335651	0.30335651	0.18393899	0.06452148
64	0.05661594	0.18393653	0.31125712	0.31125715	0.18393658	0.05661601
128	0.05457118	0.18394479	0.31331839	0.31331836	0.18394472	0.05457110
256	0.05407447	0.18393318	0.31385834	0.31385863	0.18396692	0.05407518
512	0.05392663	0.18393549	0.31405060	0.31405084	0.18394405	0.05392761
1024	0.05386625	0.18393840	0.31400978	0.31400981	0.18394110	0.05388334
*u*_exact_(*x*,1)	0.05387469	0.18393972	0.31400474	0.31400474	0.18393972	0.05387469

**Table 3:** Some numerical solution of 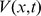 at 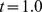.

*n*/*x*	0.125	0.25	0.375	0.625	0.75	0.875
16	0.27700610	0.18393970	0.09087339	0.09087339	0.18393970	0.27700601
32	0.30361313	0.18393970	0.06426627	0.06426627	0.18393970	0.30361313
64	0.31132848	0.18393971	0.05665094	0.05665093	0.18393969	0.31132845
128	0.31333062	0.18393969	0.05454876	0.05454877	0.18393971	0.31333065
256	0.31383601	0.18393980	0.05404358	0.05404347	0.18393960	0.31383575
512	0.31396262	0.18393980	0.05391696	0.05391688	0.18393963	0.31396241
1024	0.31399647	0.18394147	0.05388465	0.05388429	0.18393793	0.31399625
*v*_exact_(*x*,1)	0.31400474	0.18393972	0.05387469	0.05387469	0.18393972	0.31400474

**Figure 1:**
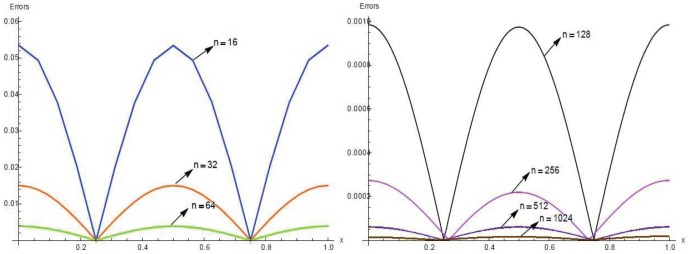
The absolute error curves of numerical solution 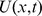 at 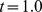.

**Figure 2:**
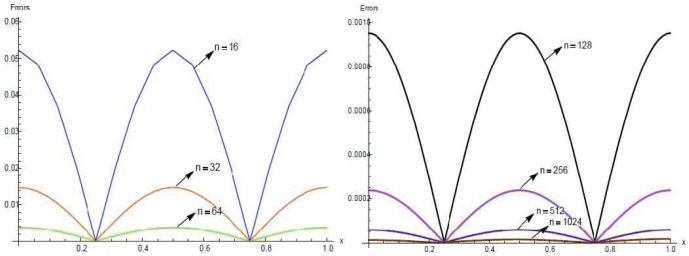
The absolute error curves of numerical solution 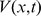 at 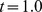.

This problem is tested using different values of 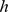 and 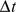 to show the capability of the proposed method for solving the system (21)–(23). The final time is taken as 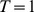. The maximum absolute errors of the method at some grid points are comparable with finite difference scheme in [1]. The numerical errors of the proposed method are presented in Table 4 and Table 5 and are also depicted graphically in Figure 1 and Figure 2. The absolute errors of the numerical solution using finite difference scheme in [1] are shown in Table 6 and Table 7

**Table 4:** The absolute errors at different grid points of numerical solution of 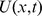 at 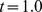.

n/x	0.125	0.25	0.375	0.625	0.75	0.875
16	3.7790E-02	5.3228E-07	3.7791E-02	3.7791E-02	5.3354E-07	3.7790E-02
32	1.0646E-02	7.2246E-07	1.0648E-02	1.0648E-02	7.2208E-07	1.0646E-02
64	2.7412E-03	3.1889E-06	2.7476E-03	2.7476E-03	3.1346E-06	2.7413E-03
128	6.9649E-04	5.0748E-06	6.8634E-04	6.8638E-04	5.0093E-06	6.9640E-04
256	1.9977E-04	6.5450E-06	1.4640E-04	1.4610E-04	2.7209E-05	2.0048E-04
512	5.1944E-05	4.2320E-06	4.5862E-05	4.6101E-05	4.3318E-06	5.2920E-05
1024	8.4430E-06	1.3219E-06	5.0421E-06	5.0741E-06	1.3811E-06	8.6510E-06

**Table 5:** The absolute errors at different grid points of numerical solution of 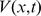 at 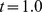.

n/x	0.125	0.25	0.375	0.625	0.75	0.875
16	3.6998E-02	1.5559E-08	3.6998E-02	3.6998E-02	1.5097E-08	3.6998E-02
32	1.0391E-02	1.5257E-08	1.0391E-02	1.0391E-02	1.5398E-08	1.0391E-02
64	2.6762E-03	5.3785E-09	2.6762E-03	2.6762E-03	2.5277E-08	2.6762E-03
128	6.7411E-04	2.7316E-08	6.7406E-04	6.7407E-04	3.3408E-09	6.7408E-04
256	1.6873E-04	8.3759E-08	1.6888E-04	1.6877E-04	1.1441E-07	1.6898E-04
512	4.2123E-05	8.0580E-08	4.2271E-05	4.2184E-05	8.0882E-08	4.2334E-05
1024	8.2681E-06	1.7575E-06	9.9601E-06	9.5943E-06	1.7881E-06	8.4926E-06

**Table 6:** The absolute errors of numerical solution [1] of 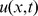 at 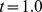.

*n*/*x*	0.125	0.375	0.625	0.875
16	1.3906E-03	9.7932E-04	9.7932E-04	1.3906E-03
32	3.4719E-04	2.4432E-04	2.4432E-04	3.4719E-04
64	8.6706E-05	6.0986E-05	6.0986E-05	8.6706E-05
128	2.1670E-05	1.5240E-05	1.5240E-05	2.1670E-05
256	5.4173E-06	3.8098E-06	3.8098E-06	5.4173E-06
512	1.3543E-06	9.5242E-07	9.5242E-07	1.3543E-06

**Table 7:** The absolute errors of numerical solution [1] of 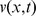 at 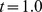.

*n*/*x*	0.125	0.375	0.625	0.875
16	1.1016E-03	1.5129E-03	1.5129E-03	1.1016E-03
32	2.6765E-04	3.7052E-04	3.7052E-04	2.6765E-04
64	6.6834E-05	9.2554E-05	9.2554E-05	6.6834E-05
128	1.6703E-05	2.3133E-05	2.3133E-05	1.6703E-05
256	4.1756E-06	5.7831E-06	5.7831E-06	4.1756E-06
512	1.0438E-06	1.4457E-06	1.4457E-06	1.0438E-06

**Figure 3:**
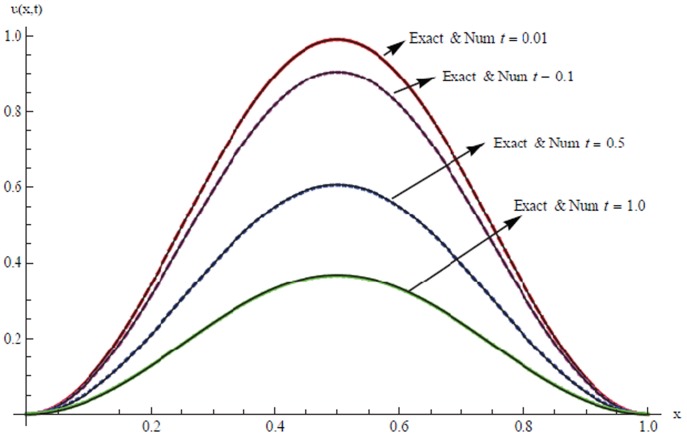
A comparison between numerical 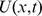 and exact solutions at different time levels.

**Figure 4:**
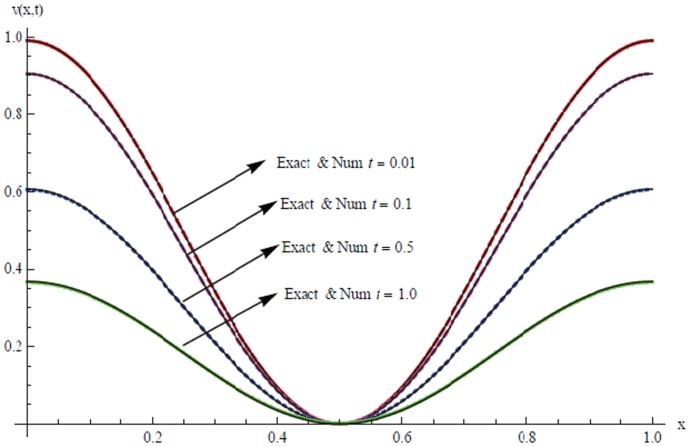
A comparison between numerical 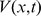 and exact solutions at different time levels.

The solutions are also tabulated in Table 8 and Table 9 with different number of partition and different time levels. Results are presented graphically for 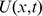 and 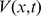 in Figure 3 and Figure 4 for 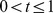 respectively.

**Table 8:** Errors at different time-levels for 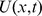 with 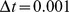.

*t*	*n* = 100 *L*_2_	*L*_∞_	*n* = 300 *L*_2_	*L*_∞_	*n* = 500 *L*_2_	*L*_∞_
0.01	5.90E-05	6.95E-05	6.56E-06	7.67E-06	2.36E-06	2.76E-06
0.1	4.24E-04	5.47E-04	4.71E-05	6.04E-05	1.69E-05	2.17E-05
0.5	1.33E-03	2.56E-03	1.48E-04	2.83E-04	5.34E-05	1.01E-05
1.0	1.60E-03	5.06E-03	1.81E-03	5.61E-03	8.10E-05	2.15E-05

**Table 9:** Errors at different time-levels for 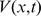 with 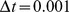.

*t*	*n* = 100 *L*_2_	*L*_∞_	*n* = 300 *L*_2_	*L*_∞_	*n* = 500 *L*_2_	*L*_∞_
0.01	1.05E-05	1.22E-05	1.16E-06	1.36E-06	4.21E-07	4.90E-07
0.1	3.25E-04	4.14E-04	3.62E-05	4.61E-05	1.30E-05	1.66E-05
0.5	1.26E-03	2.40E-03	1.40E-04	2.68E-04	5.07E-05	9.65E-05
1.0	1.55E-03	4.87E-03	1.74E-04	5.45E-04	6.39E-05	1.96E-05

The order of convergence of the present example is calculated by the use of the formula given in (20) and which is tabulated in Table 10 and Table 11 for 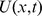 and 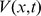 respectively. An examination of these tables indicates the method has a nearly second order of convergence.

**Table 10:** Maximum error, ratio and order of convergence of the proposed scheme for 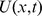.

*t* = 0.1 *n*	*L*_∞_	Ratio	Order of Conver.	*t* = 0.5 *L*_∞_	Ratio	Order of Conver.
16	1.64E-02	..........	...........	4.8E-02	..........	...........
32	4.13E-03	3.9623	1.9863	1.27E-02	3.7704	1.9147
64	1.04E-03	3.9906	1.9966	3.24E-03	3.9408	1.9785
128	2.59E-04	3.9975	1.9991	8.13E-04	3.9850	1.9945
256	6.48E-05	3.9990	1.9996	2.03E-04	3.9959	1.9985
512	1.62E-05	3.9984	1.9994	5.09E-05	3.9983	1.9994
1024	4.06E-06	3.9973	1.9979	1.27E-05	3.9868	1.9952

**Table 11:** Maximum error, ratio and order of convergence of the proposed scheme for 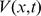.

*t* = 0.1 *n*	*L*_∞_	Ratio	Order of Conver.	*t* = 0.5 *L*_∞_	Ratio	Order of Conver.
16	1.26E-02	...........	...........	4.60E-02	............	............
32	3.17E-03	3.9819	1.9834	1.21E-02	3.7833	1.9196
64	7.95E-04	3.9956	1.9984	3.08E-03	3.9443	1.9797
128	2.59E-04	3.9988	1.9995	7.73E-04	3.9859	1.9949
256	4.97E-05	3.9992	1.9997	1.93E-04	3.9961	1.9985
512	1.24E-05	3.9981	1.9993	4.84E-05	3.9975	1.9991
1024	3.11E-06	3.9927	1.9973	1.21E-05	3.9933	1.9975

## Concluding Remarks

In this paper, a numerical method which incorporates a usual finite difference scheme with cubic B-spline is presented for solving the strongly coupled reaction diffusion system. A finite difference approach is used to discretize the time derivatives and cubic B-spline is used to interpolate the solutions at each time level. It is noted that sometimes the accuracy of solution reduces as time increases due to the time truncation errors of time derivative term [19]. However the cubic B-spline method used in this work is simple and straight forward to apply. The computed results show that the cubic B-spline gives reasonable solutions which are comparable with finite difference scheme with smaller space steps. The obtained solution to the reaction diffusion system for various time levels have been compared with the exact solution by finding the 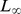 and 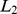 errors. An advantage of using the cubic B-spline method outlined in this paper is that it can give accurate solutions at any intermediate point in the space direction.
